# New insights into experimental visceral leishmaniasis: Real-time *in vivo* imaging of *Leishmania donovani* virulence

**DOI:** 10.1371/journal.pntd.0005924

**Published:** 2017-09-25

**Authors:** Guilherme D. Melo, Sophie Goyard, Hervé Lecoeur, Eline Rouault, Pascale Pescher, Laurence Fiette, Alexandre Boissonnas, Paola Minoprio, Thierry Lang

**Affiliations:** 1 Institut Pasteur, Laboratoire des Processus Infectieux à Trypanosomatidés, Département Infection et Epidémiologie, 25–28 rue du Dr Roux, Paris, France; 2 Institut Pasteur, Unité de Parasitologie Moléculaire et Signalisation, Département de Parasites et Insectes Vecteurs, 25–28 rue du Dr Roux, Paris, France; 3 Institut Pasteur, Unité d’Histopathologie Humaine et Modèles Animaux, Département Infection et Epidémiologie, 25–28 rue du Dr Roux, Paris, France; 4 Sorbonne Universités, UPMC Univ Paris 06, Inserm, UMR 1135, CNRS, ERL 8255, Centre d’Immunologie et des Maladies Infectieuses (CIMI-Paris), 91 Boulevard de l'Hôpital, Paris, France; Pasteur Institute of Iran, ISLAMIC REPUBLIC OF IRAN

## Abstract

Visceral leishmaniasis is an insidious neglected disease with worldwide distribution. It is caused by parasites from the *Leishmania donovani* complex, which are able to be transmitted by different species of phlebotomine sand flies and to infect numerous mammal hosts. Despite the high number of people infected or at risk, and the remarkable quantity of studies focusing on this disease, a proper experimental model to efficiently decipher the infectious process of visceral leishmaniasis taking into account the nuances of parasite’s virulence and the duration of the infection is still lacking. Therefore, using golden Syrian hamsters and BALB/c mice, state-of-the-art genetic manipulation applied on a fully virulent *L*. *donovani* strain and *in vivo* imaging approaches, we describe herein three benefits for experimental visceral leishmaniasis: (i) the development of a double transfected bioluminescent (firefly luciferase) and fluorescent (E2-crimson) virulent strain of *L*. *donovani* (Ld1S_luci_E2-crimson), favoring a wide range of both *in vivo* and *in vitro* investigations, (ii) the establishment of a non-invasive mouse model to evaluate the infectious process during visceral leishmaniasis and the parasite’s virulence in real time, allowing longitudinal studies with the same animals, and (iii) the elaboration of a suitable method to reinstate (and verify anew) the virulence in a population of attenuated parasites, by recovering persistent parasites from chronic infected mice. Consequently, these results open up new perspectives on the study of visceral leishmaniasis, especially in the fields of therapeutics and vaccinology, since the model described herein renders now possible long-lasting follow up studies, with easy and accurate day-by-day verifications of the infection status along with a reduced number of laboratory animals.

**Trial registration:**

ClinicalTrials.gov 2013-0047.

## Author summary

Visceral leishmaniasis is a chronic disease caused by the life-threatening parasite *Leishmania donovani*. This chronicity character is a major concern in experimental models of visceral leishmaniasis, since infected animals do not develop any visible sign or symptom before one/two months after infection. Consequently, there is an urge to develop a model that allow the early identification of the parasite in the host’s tissues and an easy follow up of the infection. Therefore, we generated genetically-modified virulent parasites that are concomitantly fluorescent and bioluminescent, and using highly performant imaging approaches, we could detect these parasites in tissues of infected hamsters and mice within a week post-infection. With these parasites, we established, then, a mouse model of visceral leishmaniasis that allows prolonged longitudinal studies and enables the investigation of parasite’s survival, using non-invasive *in vivo* imaging techniques. These features are of extreme importance, particularly for the development of new therapies and vaccines and open up new perspectives on the study of this disease.

## Introduction

Protozoan parasites of the genus *Leishmania* are the causative agents of leishmaniasis, a neglected disease with worldwide distribution. With 350 million people at risk and 300 000 estimated cases, the visceral form of leishmaniasis (VL) can be fatal if not treated. The symptoms could vary from fever, weight loss and anorexia to cachexia, hepato/splenomegaly, lymphadenopathy, renal injuries, hemorrhages [1,2].

The perpetuation of these parasite populations relies on the subversion of their two hosts: blood-feeding insects and mammals. The establishment of parasites in tissues, including blood, liver, bone marrow, spleen, skin, depends on their capacity to weaken their hosts by parasite-specific and non-specific mechanisms [1,3]. The thorough study of the events occurring in the mammalian hosts that are relevant for infectivity and persistence may contribute to a better understanding of parasite dissemination mechanisms and pathogenesis, leading to novel therapeutic strategies.

Few *in vivo* models have been proposed to study VL, with dogs and hamsters being the most reliable [4–6], since the experimentally infected animals exhibit similar clinicopathological aspects as VL in humans. However, restrictions regarding the number of animals, the prolonged time of infection needed to observe clinical signs and to obtain high parasite loads, the high costs, besides ethical issues, make necessary the search for other suitable alternatives. Regarding the experimental models of VL in mice, the studies depict a premature control of the parasite load in the liver and a delayed parasite burden in the spleen [7–9], rendering difficult a global long-term evaluation of parasite persistence. All these data indicate a critical need to set up an efficient and functional experimental animal model for VL using virulent parasites. Such model should allow the real-time evaluation of the parasite load and the infectious process in living animals, which must be sensitive enough to assess their pathogenesis and persistence in mammals.

We and others have previously demonstrated that the use of imaging is exceptionally helpful for visualizing the dynamic processes that take place in tissues and examining host’s cell-parasite interaction events. So far, different species of bioluminescent or red-fluorescent *Leishmania* parasites have been constructed and used for *in vivo* imaging [10–19]. Recently, imaging VL in real time with the golden hamster model has been proposed using bioluminescent and virulent *Leishmania donovani* [20,21], however, few imaging studies have been proposed to analyze the dynamic of the VL infectious process and parasite virulence [8,11,22].

Therefore, to overcome the limits of the mouse models of VL, with low number of parasites and low persistence, we used a bioluminescent and fluorescent virulent strain of *L*. *donovani*, that would easily allow their follow up both *in vivo* and *in vitro*, and which should be able to induce a persistent infection in BALB/c mice. We took advantage of highly performant imaging approaches to follow the parasite load in the liver and in the spleen of infected mice together with the evaluation of cytokine gene expression in these organs. The ability of attenuated bioluminescent/fluorescent *L*. *donovani*, generated after serial long-term *in vitro* culturing, for implantation and persistence in the target organs and whether the persistent parasites are still able to promote infection was also assessed. Our study provides a basis for determining the parasite implantation and dissemination during the disease progression *in vivo* in a very useful functional model, allowing to assess the role of candidate targets in parasites viability and virulence in order to evaluate their potential putative druggability.

## Methods

### Animals

Female golden Syrian hamsters (*Mesocricetus auratus* RjHan:AURA) weighing 60–70 g and six-week-old female BALB/c ByJRj mice were purchased from Janvier Laboratories (Le Genest-Saint-Isle, France), and handled under specific pathogen-free conditions, according to the institutional guidelines of the Central Animal Facility at Pasteur Institute (Paris, France).

### Parasites

A fully virulent *Leishmania donovani* strain (Ld1S/MHOM/SD/00-strain 1S) obtained from infected hamster spleen was used in this study. The culture medium used was the M199 supplemented with 25 mM of HEPES pH6.9; 2mM of glutamine; 0.1 of mM adenosine; 100 μg/mL of penicillin/streptomycin; 10 μg/mL of folic acid; 5 μg/mL of hemin; 1 μg/mL of biopterin; 10% heat-inactivated fetal calf serum (29-101-54, MP Biomedicals, Santa Ana, CA, USA); 7.5% of NaHCO_3_ and 1x RPMI 1640 vitamin mix.

### Amastigote collection

Infected hamsters and mice were euthanized, the spleens were collected in gentleMACS M Tubes (130-096-335, Miltenyi Biotec, Bergisch Gladbach, Germany) containing 5 mL of supplemented M199 medium and homogenized using the gentleMACS dissociator (Miltenyi Biotec). After centrifugation (800 rpm for 5 min at 20°C), 25 mg of saponin (S2149, Sigma-Aldrich, Saint-Quentin Fallavier, France) was added to the supernatant, re-centrifuged (3500 rpm for 10 min at 20°C), and washed with M199 medium. The solution was passed several times through a 27G needle connected to a syringe to improve amastigotes release. The number of amastigotes was determined and they were kept at 26°C in supplemented M199 medium (starting from 1x10^5^ amastigotes/mL) to promote differentiation into promastigotes and expansion.

### Infection

Short-term cultures of *L*. *donovani* promastigotes were obtained from splenic amastigotes. The parasites were expanded until stationary phase cultures (+2 days after the end of the exponential growth phase). Parasites were centrifuged at 2500 rpm for 5 min at 20°C, and less dense parasites, morphologically similar to metacyclic forms [23] were recovered from the supernatant by centrifugation at 4000 rpm for 10 min. Mice were infected with a standardized dose of 5x10^7^ enriched ‘metacyclic’ promastigotes in 150 μL of PBS by intraperitoneal route, and hamsters were infected with a standardized dose of 1x10^8^ enriched ‘metacyclic’ promastigotes in 200 μL of PBS by intracardiac route.

### Parasite transfection

We performed a two-step transfection to generate both bioluminescent and fluorescent parasites. For the first transfection, the luciferase coding region was cloned into the *Leishmania* expression vector pF4X1.HYG (Jenabioscience, Jena, Germany) [11]. A total amount of 5x10^7^ log-phase wild-type promastigotes was mixed with 100 ng of the linearized plasmid and to 99 μL of transfection mix (human T-cell nucleofector, VPA-1002, Lonza, Basel Switzerland). The mix was added to the AMAXA Nucleofector 2b Device (Lonza) and the transfection was performed using the pre-defined X-014 program according to the manufacturer’s instructions. The solution was then plated on a M199 solid medium supplemented with 150 μg/mL of hygromycin and cultivated at 26°C up to 14 days. Once the colonies were identified, they were collected into flasks containing M199 medium and kept at 26°C to promote promastigote expansion until infectious metacyclic promastigotes from stationary phase cultures. One hamster was infected with 1x10^8^ parasites and we followed the infection using *in vivo* bioluminescence imaging. The weight gain of the hamster was followed up weekly. Three months after infection, the splenic amastigotes (Ld1S_luci) were collected and transformed in promastigotes for a novel transfection.

The second transfection was performed as described above, but using the *Leishmania* expression vector pF4X1.SAT (Jenabioscience) containing the E2-crimson coding region, and 50 μg/mL of nourseothricin as selection drug. After transfections, the luciferase and E2-crimson genes were integrated into 18 s rRNA locus of the nuclear DNA of the parasites [11]. One hamster was infected with the transfected parasites, splenic amastigotes were then collected, transformed in promastigotes and expanded as the first passage (P1) of the parasite “Ld1S_luci_E2-crimson”.

### *In vivo* bioluminescence imaging

At different time points following *Leishmania* inoculation, D-luciferin (122799, PerkinElmer, Walthan, MA, USA), the luciferase substrate, was injected intraperitoneally in the mice with a dose of 150 mg/kg; the animals were anaesthetized in a 2.5% isoflurane atmosphere (Aerane, Baxter SA, Maurepas, France) and placed in the imaging chamber of the IVIS Spectrum (PerkinElmer). 2D-bioluminescence images were captured and total photon emission, expressed in photons/s, was determined in a region of interest (ROI: liver or spleen) using the Living Image software (PerkinElmer).

### *In vitro* characterization of Ld1S_luci_E2-crimson

The initial concentration of the *in vitro* cultures was 5x10^5^ parasites/mL. The cultures were counted on a daily-basis to obtain the growth curve and bioluminescence and fluorescence values were recorded. For concomitant acquisition of bioluminescence and fluorescence data, parasites were collected from the culture flasks, washed in PBS and 100 μL containing 1x10^5^ parasites were added to a 96-wells white plate (353377, Corning, Wiesbaden, Germany). 60 μg of D-luciferin was then added to each well. After 10 minutes of incubation, the bioluminescence and fluorescence values were consecutively determined in a TECAN luminometer (Infinity F200 Pro, TECAN, Lyon, France), using an integration time of 1000 ms at 26°C for bioluminescence, and excitation of 590 nm and emission of 670 nm for fluorescence.

### *In vivo* characterization of Ld1S_luci_E2-crimson infectious process in BALB/c mice

P1 promastigotes were injected in twelve BALB/c mice and the infection was followed using *in vivo* bioluminescence imaging, as described above, at days 7, 30, 60 and 90 post-infection (p.i.). At each time point, three infected mice were euthanized and the liver and spleen were collected for determination of the parasite load by RT-qPCR and for histology. Three uninfected mice were used as control. A total of 50 mice divided in four independent infections were used.

For RT-qPCR, livers and spleens were removed, disrupted and lysed in 5 and 3 mL of Trizol (Invitrogen, Paisley, UK), respectively, using gentleMACS M Tubes and the gentleMACS dissociator (Miltenyi Biotec). RNA isolation was performed on the clear upper aqueous layer with the RNeasy Plus Mini kit (74134, Qiagen, Courtaboeuf, France) according the manufacturer’s instructions. Evaluation of RNA quality was performed by optical density measurement using the NanoDrop spectrophotometer (Thermo Scientific, Wilmington, DE, USA) and their integrity were assessed using 2100 Bioanalyzer (Agilent Technologies, Santa Clara, CA, USA), which allowed the calculation of an RNA integrity (RNAi) number [24]. Total RNAs were reverse transcribed to first strand cDNA using random hexamers (11034731001, Roche Diagnostics, Meylan, France), a set of dNTPs (10297–018, Invitrogen) and Moloney Murine Leukemia Virus-Reverse Transcriptase (MMLV-RT, 28025013, Invitrogen).

The *Leishmania* gene target (*ssrRNA*) was selected for quantifying the number of parasites as previously described on murine cDNAs [25]. qPCR was performed in a final volume of 11 μL in 384-well PCR plates using a thermocycler (7900HT fast real time PCR system, Applied Biosystems, Villebon-sur-Yvette France). Briefly, 2 μL of cDNA (20ng) was added to 9 μL of a master mix containing 5 μl of QuantiTect SYBR Green Kit (Qiagen) and 4 μL of nuclease-free water with 1 μM of each primer (Table 1). The amplification conditions were as follows: 95°C for 15 min, 45 cycles of 95°C for 10 s, 54°C for 25 s and 72°C for 30 s; followed by a melt curve, from 60°C to 95°C.

**Table 1:** Primers sequences used for RT-qPCR and reaction efficiency values (E) for each target.

Target	Forward (5’– 3’)	Reverse (5’– 3’)	E
*Leishmania* ssrRNA	CCATGTCGGATTTGGT	CGAAACGGTAGCCTAGAG	2.04
IFN-γ	CTTCTTCAGCAACAGCAAGG	TGAGCTCATTGAATGCTTGG	1.83
IL-1β	AGGCAGGCAGTATCAC	CACACCAGCAGGTTATC	1.84
IL-2	AGGAACCTGAAACTCCC	AGTCCACCACAGTTGC	2.04
IL-4	GGAGCCATATCCACGG	AAGCCCTACAGACGAG	1.88
IL-10	CTGGACAACATACTGCTAACCGAC	ATTCATTCATGGCCTTGTAGACACC	1.99
IL-12p35	GGCCACCCTTGCCCTCCTA	GGGCAGGCAGCTCCCTCTT	1.91
TNF-α	CATCAGTTCTATGGCCC	GTGAGGAGCACGTAGT	1.92
L19	TACTGCCAATGCTCGG	AACACATTCCCTTTGACC	1.93
LDHA	AACCCTCAAGGACCAG	CAAGCTCATCCGCCAA	2.21
RPIIE	AAGATCCGCAAGACGA	GGGAAGAACACAAACATCTG	2.04
TBP	CCTATGACCCCTATCACT	GTCCGTGGCTCTCTTAT	1.86

To quantify the parasite load in each organ, serial 10-fold dilutions of parasites (from 10^8^ to 10^3^) were added either to livers or spleens recovered from naïve mice, in order to mimicry possible errors and interferences during parasites RNA extraction from the infected organs. Total RNAs were then extracted and processed for RT-qPCR as described above. A linear regression for each standard curve was determined (quantification of *Leishmania* parasites against the relative expression of *ssrRNA* values). To visualize the parasites in the tissues, liver and spleen were collected and embedded in paraffin. Four μm–thick sections were stained with hematoxylin and eosin (HE) and *Leishmania* parasites were immunolabelled on a Bond III immunostainer (Leica, Germany) using a rabbit polyclonal antibody raised against *L*. *donovani* (1:3500).

The gene expression of selected cytokines was quantified by RT-qPCR to evaluate host inflammatory mediators’ transcripts in the liver and spleen (Table 1) as described above. For normalization calculations, the pair of control genes ldha (lactate dehydrogenase A)/l19 (ribosomal protein L19) and tbp (TATA box-binding protein)/rpIIe (RNA polymerase E) were selected as the most stable reference genes for the liver and spleen of mice, respectively.

We also evaluated the presence of infected cells in the liver and in the spleen at early time points post-infection (7 days p.i.) by flow cytometry. The organs were collected and dissociated in 5 mL of PBS containing 2mM of EDTA and 0.5% (v/v) of fetal calf serum (MP Biomedicals) using gentleMACS M Tubes and the gentleMACS dissociator (Miltenyi Biotec). The cell suspension was centrifuged at 300 × g for 10 min at 4°C and the pellet was resuspended in 2 mL of PBS-EDTA. 200 μL of the cell suspension was placed in a 96-wells plate, centrifuged at 300 × g for 5 min at 4°C. The supernatant was removed and 50 μL of an anti-CD45 antibody (clone 30-F11) (Becton Dickinson) was added and incubated for 20 min at 4°C. The cells were washed in PBS and the red blood cells were lysed by washing three times with ACK (Ammonium-Chloride-Potassium) Lysing Buffer. The plate was centrifuged and the cells were resuspended in 4% (w/v) of paraformaldehyde. Flow cytometry was performed with the flow cytometer Fortessa-X20 (Becton Dickinson) and DIVA Flow Cytometry acquisition software and was analyzed with FlowJo software (Tree Star, Inc).

### Effect of successive *in vitro* passages on parasite infectivity

Promastigotes freshly derived from splenic amastigotes kept in M199 medium were subjected to a weekly *in vitro* passage, with the initial concentration of 5x10^5^ parasites/mL. A total of 41 weekly passages was performed. Parasites at passages P5 (five weeks *in vitro*), P11 (eleven weeks *in vitro*), P21 (twenty-one weeks *in vitro*) and P41 (forty-one weeks *in vitro*) were selected, and data regarding bioluminescence, fluorescence and doubling time were recorded. Parasites obtained at passages P5, P11, P21 and P41 were expanded until stationary phase for promastigotes enrichment as previously described, and injected into BALB/c mice (seven animals/passage). *In vivo* analyses of the infected mice were performed during 90 days as described above.

### Restoration of parasite infectivity: Effect of *in vivo* passages in mouse spleen

Amastigotes from the spleen of mice infected with P1 and P21 parasites were collected at day 90 p.i. as described above. Enriched ‘metacyclic’ promastigotes derived from mice P1 (named mP1) and from mice P21 (named mP21) were then injected into seven naïve BALB/c mice, which were followed up during 90 days p.i.. *In vivo* analyses of the infected mice were performed as described above.

### Statistical analyses

The comparison between the groups was performed by Kurskal-Wallis followed by Dunn’s multiple comparison test, or by Mann Whitney test. The area under the curve (AUC) was used to compare the infection kinetics. Values of P<0.05 were considered statistically significant. Data were expressed as the median and the interquartile range. For qPCR, the variations in the gene-expression were calculated as the n-fold change in expression in the organs of the infected mice compared to the organs of the uninfected ones. The relative expression software tool (*REST-MCS)* was used to determine group wise comparison and statistical analysis of relative expression results in real-time PCR [26].

### Ethical statement

All animal experiments were conducted in accordance with the project registered under number 2013–0047 and approved by the Institut Pasteur Ethics Committee (CETEA) on November 12, 2014 in accordance to the European legislation/guidelines EU 2010/63.

## Results

### Double transfection do not change Ld1S parasites performance

**Fig 1:**
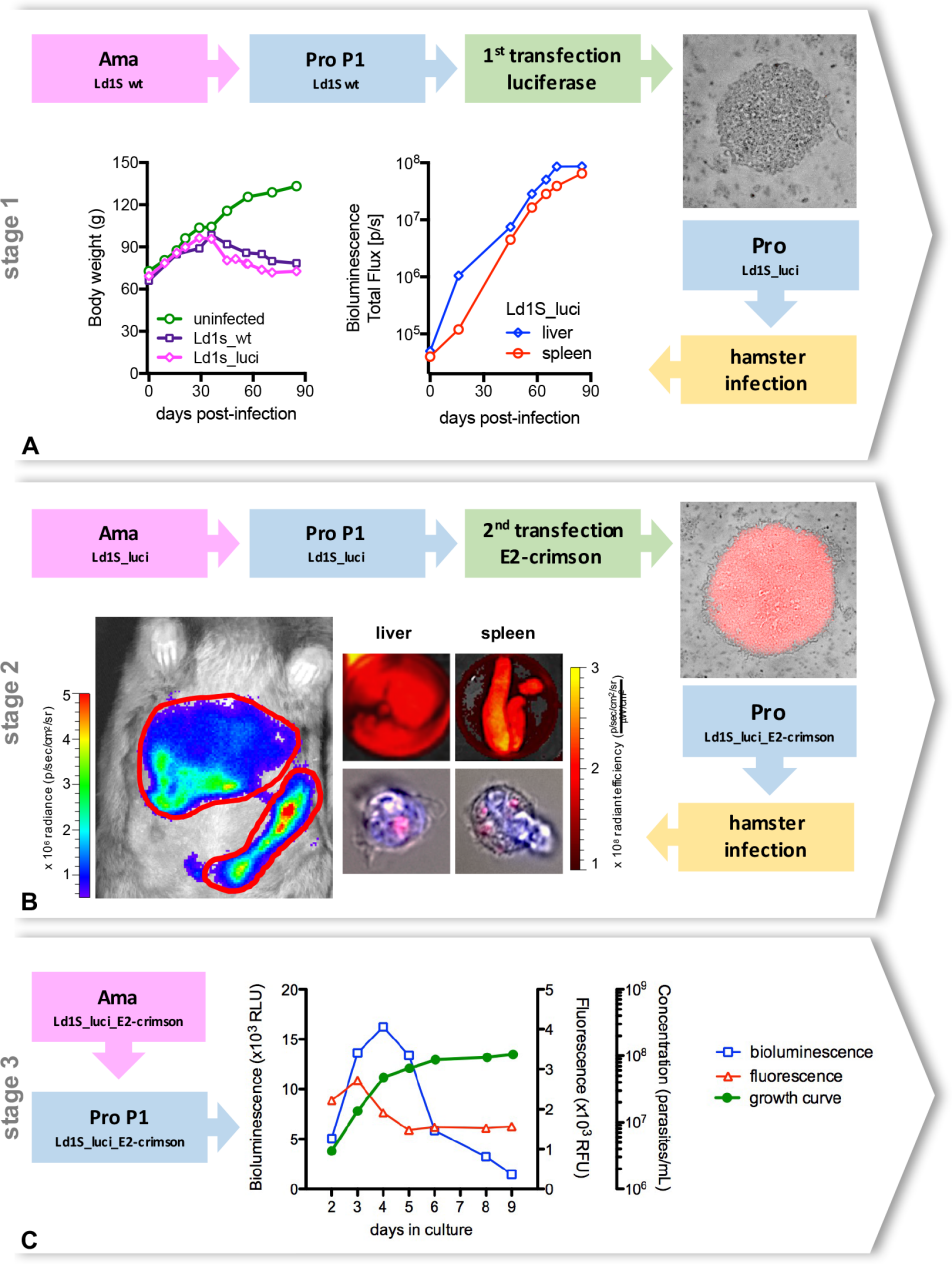
Generation of double transfected *Leishmania donovani* parasites. (**A**) The first stage of the transfection aimed to generate bioluminescent parasites. Wild-type amastigotes from the Ld1S strain obtained from hamster spleen were transformed into promastigotes, then transfected with the luciferase gene and cloned in M199-agar dishes containing 150 **μ**g/mL hygromycin. Positive colonies (top right corner) were selected, added to liquid M199 medium and promastigotes were cultivated until differentiation into metacyclic promastigotes in order to proceed with a hamster infection. Weight gain of the hamster infected with ‘Ld1S_luci’, in comparison with an uninfected hamster and with a hamster infected with wild-type amastigotes (Ld1s_wt) (bottom left). *In vivo* bioluminescence values in the liver and in the spleen of the hamster infected with ‘Ld1S_luci’ (bottom middle). (**B**) The second stage of the transfection aimed to generate fluorescent parasites. ‘Ld1S_luci’ amastigotes were obtained from the hamster spleen, transformed into promastigotes, transfected with the E2-crimson gene and cloned in M199-agar dishes containing 50 **μ**g/mL of nourseothricin. Fluorescent colonies (top right corner) were selected, added to liquid M199 medium and promastigotes were cultivated until differentiation into metacyclic promastigotes in order to proceed with a hamster infection with ‘Ld1S_luci_E2-crimson’. Hamster *in vivo* bioluminescence imaging (bottom left, and S1 Fig) and simultaneous *ex vivo* analyses of fluorescence in the liver and in the spleen at day 90 post-infection (top middle panels). Red fluorescent parasites are also detectable in the cytoplasm of isolated hamster cells (nuclei counterstained with DAPI) (bottom middle panels). (**C**) ‘Ld1S_luci_E2-crimson’ amastigotes were obtained from the hamster spleen and transformed into promastigotes, considered then the first passage of promastigotes (P1). Bioluminescence (left y-axis) and fluorescence (first right y-axis) values, as well as the growth curve (second right y-axis) obtained from P1 ‘Ld1S_luci_E2-crimson’ promastigotes cultivated *in vitro* are shown in the graph.

The double transfection approach generated bioluminescent and fluorescent Ld1S parasites as virulent as Ld1S wild-type ones. Indeed, the virulence of the transfected parasites was verified at each stage of the process. Hamsters infected with Ld1S_luci presented the same characteristics than hamsters infected with Ld1S wild-type parasites, such as a regular weight gain until 30 days post-infection, with a subsequent progressive weight loss. By measuring the bioluminescent signals in the liver and the spleen of the infected hamster, we noticed a gradual increase of the parasite load in both organs (Fig 1A).

With the second transfection, we inserted the E2-crimson gene aiming to generate fluorescent parasites. Even though bioluminescence is more adapted to *in vivo* analysis, due to its high sensitivity and low background signals, fluorescence is helpful during *in vitro* and *ex vivo* experiments [27,28]. Since the fluorescent protein is constitutively expressed by the parasites, we could see positive results as soon as the colonies of parasites were identified in the solid medium during the cloning stage after the transfection (Fig 1B). The kinetics of bioluminescence of infected hamsters with Ld1S_luci_E2-crimson remained identical to those infected with Ld1S_luci (S1 Fig), corroborating the maintenance of the parasites’ infectivity. At day 90 p.i., the time point of amastigotes collection, successive *in vivo* and *ex vivo* imaging of the liver and spleen showed concomitant positive bioluminescent and fluorescent signals in these organs (Fig 1B). Ld1S_luci_E2-crimson amastigotes were visualized in infected cells (Fig 1B), and they were collected, purified and differentiated into promastigotes during the first passage in culture, and called P1.

The behavior of the P1 Ld1S_luci_E2-crimson promastigotes was similar to the wild-type promastigotes, with a doubling time of 22 hours and a stationary phase concentration of approximately 1x10^8^ parasites/mL at day 7. The bioluminescence and fluorescence features of the parasites evidenced stage-specific changes throughout the period in culture (nine days). Bioluminescence values rose sharply during the exponential phase of the growth curve, with a peak at day 4, and then they decreased sharply as well, with ‘metacyclic’ promastigotes (day 9) presenting the smallest values. On the other hand, variations in fluorescence intensity were less pronounced, with a slight peak at day 3, and constant values after day 5 (Fig 1C).

### ‘Ld1S_luci_E2-crimson’ parasites promote a long-lasting infection and induce a typical immune response in mice

**Fig 2:**
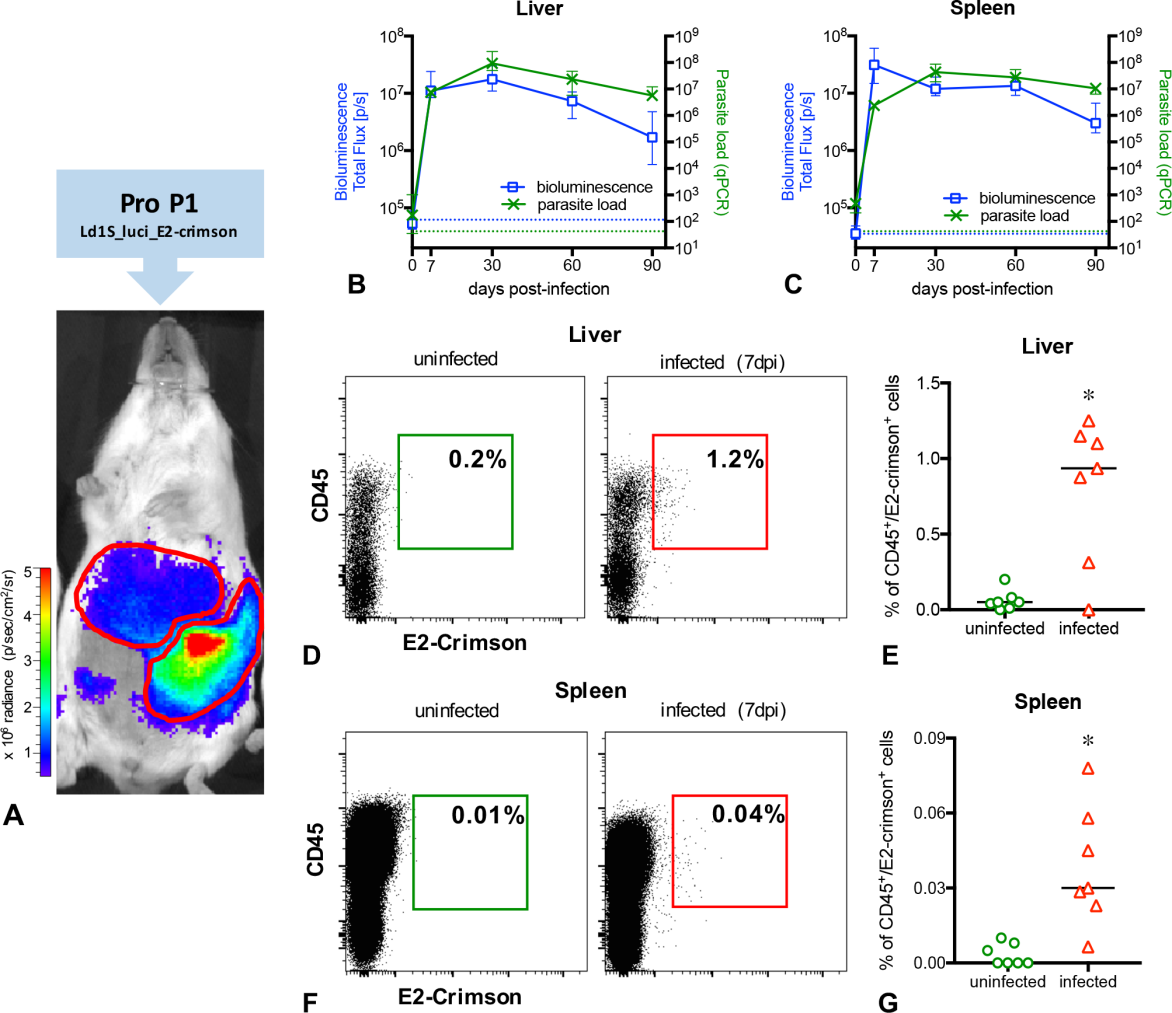
*In vivo* and *ex vivo* determination of the parasite load in BALB/c mice infected with P1 ‘Ld1S_luci_E2-crimson’. (**A**) *In vivo* imaging of a representative infected mouse exhibiting positive bioluminescent signals in the liver and in the spleen. (**BC**) Follow-up of the infection in the liver (**B**) and in the spleen (**C**) using *in vivo* bioluminescence (left y-axis) and RT-qPCR (right y-axis) at different time points post-infection. Representative data of 50 infected mice, from four independent infection groups. Data are expressed as the median and the interquartile range. The dotted blue lines correspond to background signals for bioluminescence, and the dotted green lines correspond to background values for RT-qPCR. (**DG**) Flow cytometry analysis of liver and spleen cells from mice, using the markers CD45 (leukocytes) and E2-crimson (Ld1S_luci_E2-crimson). Representative dot plots and variations of CD45^+^E2-crimson^+^ cells in the liver (**DE**) and in the spleen (**FG**) of uninfected and infected mice at day 7 p.i. (* indicates P<0.05).

**Fig 3:**
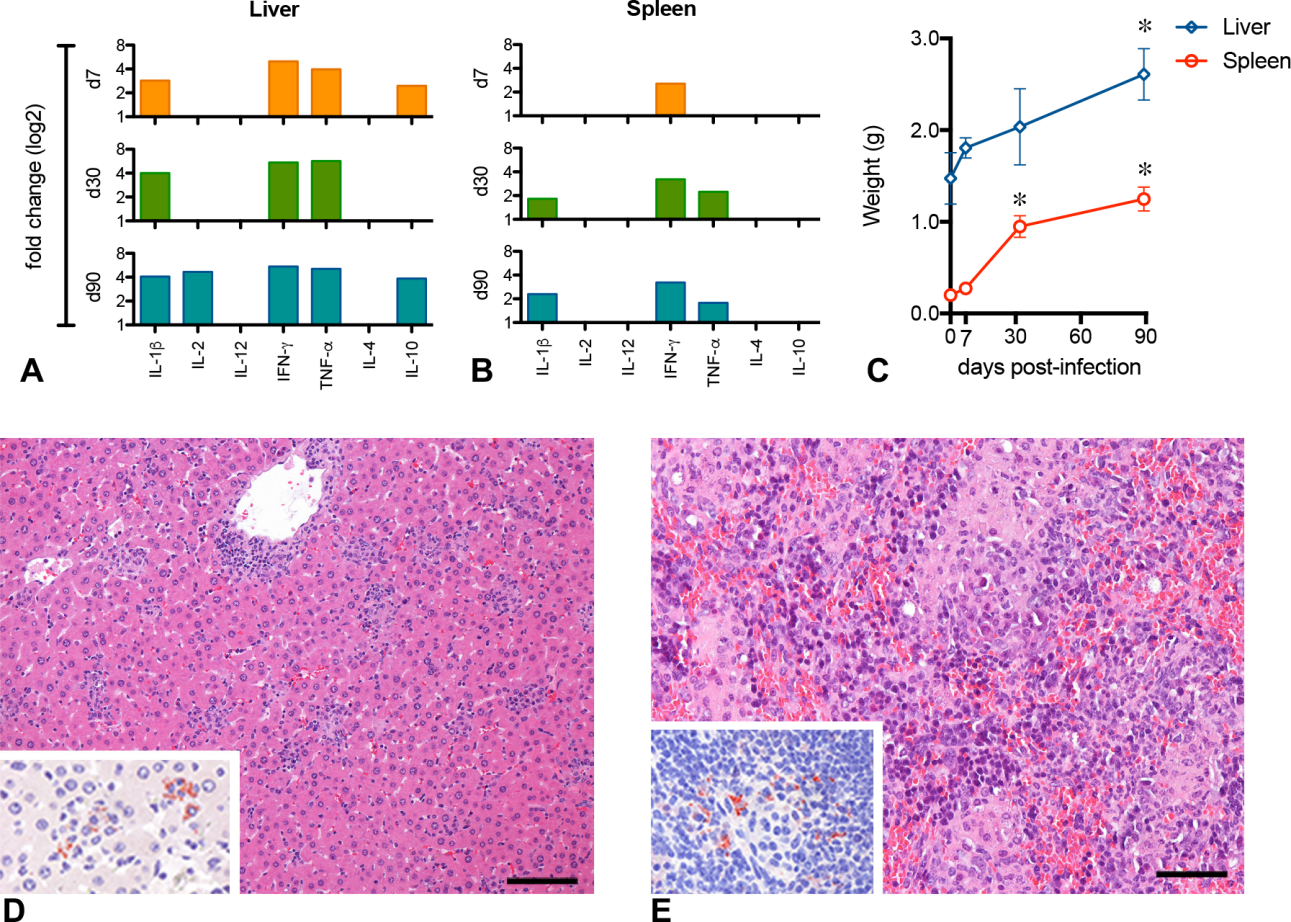
Inflammatory response in BALB/c mice infected with P1 ‘Ld1S_luci_E2-crimson’. Inflammatory response assessment in the liver (**A**) and spleen (**B**) of BALB/c mice infected with *Leishmania donovani*. The relative gene expression of cytokines in the liver and spleen is expressed as fold change (up-regulation) for each time point post-infection. Bars represent statistically significant changes (P<0.05). (**C**) Follow-up of liver and spleen weight at different time points post-infection. Data are expressed as the median and the interquartile range (* time points differ significantly in comparison to day 0; P<0.05). (**DE**) Histopathological analysis of liver and spleen at 90 days p.i. (**D**) Liver with granulomas and scattered intracellular parasites (inset; immunoperoxidase). (**E**) Spleen presenting remarkable alterations in its architecture, with an important amount of mononuclear cells infiltration and parasites (inset; immunoperoxidase). Hematoxylin and eosin. Scale bars = 100 μm.

We analyzed P1 Ld1S_luci_E2-crimson implantation and persistence in the liver and in the spleen by bioluminescent *in vivo* imaging, RT-qPCR and by flow cytometry (Fig 2); the immune response was assessed by cytokine gene expression analysis (RT-qPCR) and lesions in these organs were evaluated by histopathology (Fig 3).

In both liver and spleen, the first bioluminescent signals were detected as soon as 7 days p.i., then rose slightly to reach a peak at day 30 p.i. (Fig 2A–2C). After this, the bioluminescence signal declined slightly up to 90 days pi. To determine the absolute number of *Leishmania* in the whole organs, parasite transcripts were quantified by RT-qPCR. *L*. *donovani* transcript abundance and bioluminescence kinetics recorded from the liver and the spleen had a similar profile, indicating a good correspondence between RT-qPCR and bioluminescence (Fig 2B and 2C).

These versatile parasites were sensitive enough to be detected as soon as 7 days p.i. also by flow cytometry (Fig 2D–2G). In infected mice livers, a median value of 0.96% of CD45^+^ cells containing E2-crimson fluorescence (parasitized cells) was observed, significantly higher (P>0.0001) than the uninfected control (Fig 2E). Similarly, the median value of 0.03% of CD45^+^E2-crimson^+^ cells were detected in the spleen of infected mice, significantly higher (P>0.0023) than the uninfected control (Fig 2G).

Finally, in order to attest Ld1S_luci_E2-crimson virulence, we evaluate the immune response in infected mice by determining the gene expression of selected pro- and anti-inflammatory cytokines in the liver and in the spleen, compared to uninfected animals (Fig 3A and 3B). IFN-γ was up-regulated in both organs, throughout the study, from 7 to 90 days p.i. The response in the liver seemed to be more intense than in the spleen, with up-regulation of IL-1β, IL-2 and TNF-α, along with high levels of IL-10. The spleen, on the other hand, presented a delayed and discreet overexpression of IL-1β and TNF-α. The weight of both organs considerably increased during the infection (Fig 3C), and at histology at 90 days p.i., the liver presented typical granulomas and parasites (Fig 3D) whereas the spleen presented remarkable alterations in its architecture, with important amounts of mononuclear cells infiltration and parasites (Fig 3E).

### Serial *in vitro* passages quickly affect the overall performance of ‘Ld1S_luci_E2-crimson’ parasites

**Fig 4:**
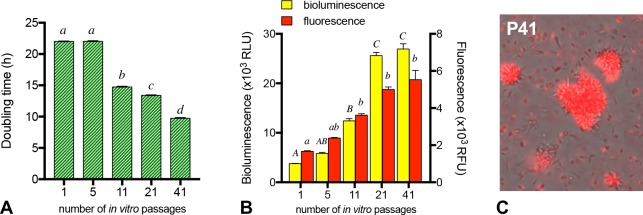
Effect of serial *in vitro* passages on *Leishmania donovani* ‘Ld1S_luci_E2-crimson’ promastigotes performance. (**A**) Doubling time at the exponential growth phase of *in vitro* cultures of different passage numbers: P1, P5, P11, P21 and P41 (^abcd^ groups with no common superscript letter differ significantly; P<0.05). (**B**) Bioluminescence and fluorescence values of 1x10^5^ parasites taken from 2-days old *in vitro* cultures of different passage numbers: P1, P5, P11, P21 and P41. Columns represent the median values and the interquartile range (^ABCabc^ groups with no common superscript capital letter differ significantly for bioluminescence, and groups with no common superscript small letter differ significantly for fluorescence; P<0.05). (**C**) Representative photomicrograph of P41 ‘Ld1S_luci_E2-crimson’ promastigotes to illustrate the constant expression of the red fluorescent protein.

Serial successive *in vitro* passages promote remarkable changes in promastigotes behavior (Fig 4A and 4B). Regarding their growth, whereas P1 and P5 presented a rather stable doubling time of around 22 hours, the speed of growth increased sharply over the passages, with a doubling time as low as 9.8 hours for P41 promastigotes (Fig 4A). Along with the speed of parasite growth measured on 1x10^5^ log-phase promastigotes, both bioluminescence and fluorescence values remarkably increased over the passages (Fig 4B). Additionally, the expression of luciferase and E2-crimson by the parasites was permanent throughout the successive *in vitro* passages (Fig 4C).

**Fig 5:**
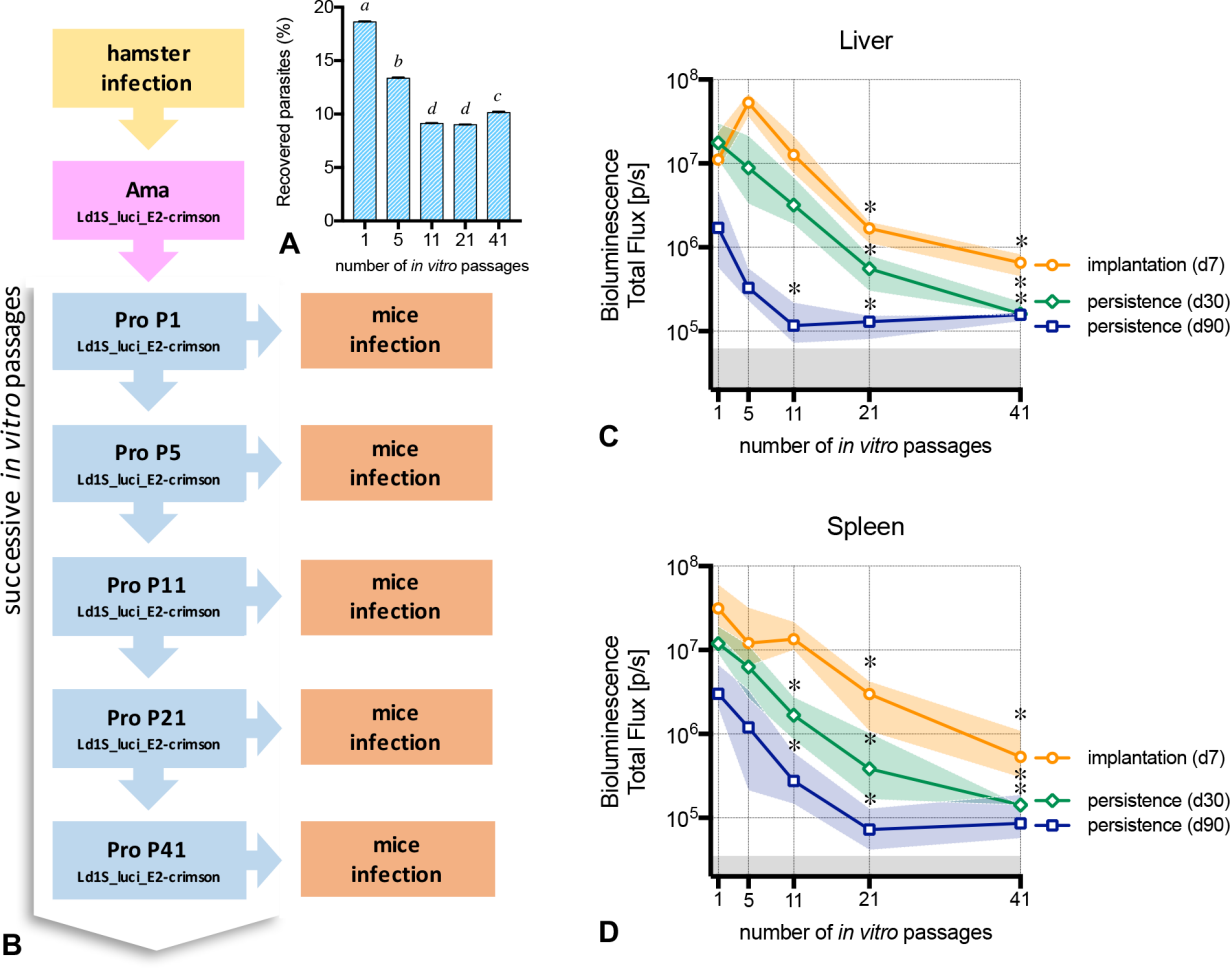
‘Ld1S_luci_E2-crimson’ infectivity after serial *in vitro* passages. (**A**) Yield of enriched ‘metacyclic’ promastigotes recovered after the end of the stationary growing-phase, used to inoculate mice (^abcd^ groups with no common superscript letter differ significantly; P<0.05). (**B**) Illustration of the sequential stages of the study. Following the collection of ‘Ld1S_luci_E2-crimson’ amastigotes from the hamster spleen, promastigotes were obtained and cultivated *in vitro*. The first *in vitro* passage of promastigotes (named P1) were used to infect mice and to start a sequence of successive *in vitro* passages of promastigotes, up to the 41st passage. At selected time points (P5, P11, P21 and P41), the promastigotes were allowed to expand until metacyclic promastigotes in order to infect mice. (**CD**) Five groups of mice were infected with enriched promastigotes obtained from five different *in vitro* passages and bioluminescence values in the liver (**C**) and in the spleen (**D**) were evaluated at day 7 p.i. in order to assess the parasite implantation, and at days 30 and 90 p.i. to verify the parasite persistence in the tissues. Lines represent the median and the colored shaded areas represent the interquartile range. The grey areas correspond to background signals (* time points differ significantly in comparison to passage 1; P<0.05).

The percentage of recovered promastigotes after ‘metacyclic’ enrichment by differential centrifugation decreased through the sequential cultures (Fig 5A). Subsequently, infection of mice with a standardized dose of 5x10^7^ enriched ‘metacyclic’ promastigotes obtained from different *in vitro* passages (Fig 5B) promoted different patterns of infection. The implantation of the parasites in the liver and spleen measured at 7 days p.i., was remarkably different, both in livers (Fig 5C) and spleens (Fig 5D). Mice infected with P1, P5 and P11 presented no significant differences, nevertheless, the parasite load of P21 and P41 was drastically lower (P<0.0001) in both organs.

To address the persistence of the parasite in the infected organs, we measured the bioluminescence signals in the liver and in the spleen at days 30 and 90 post-infection (Fig 5C and 5D). At day 30, the liver of mice infected with P21 and P41 presented lower bioluminescence values compared to P1 (P<0.0001). In the spleen, mice infected with P11, P21 and P41 presented lower bioluminescence values compared to P1 (P<0.0001). At day 90, all mice infected with P11, P21 and P41 presented a reduction in the bioluminescence in both liver (P = 0.0002) and spleen (P = 0.0005), with values close to the background signals. P5, even if not statistically different from P1, presented lower and quite heterogeneous values, especially in the spleen.

### Successive passages in mice restore ‘Ld1S_luci_E2-crimson’ infectivity lost after prolonged *in vitro* cultivation

**Fig 6:**
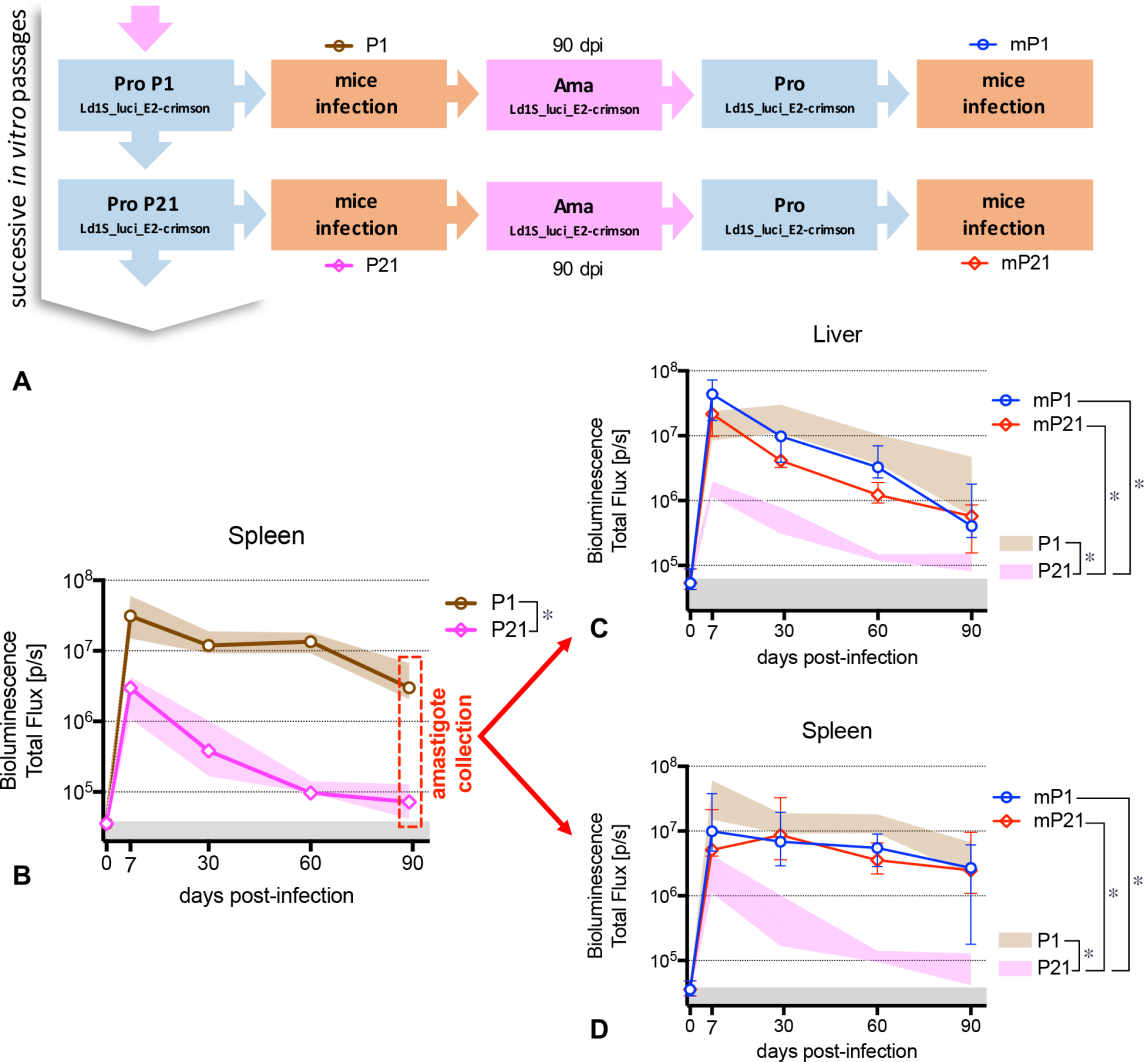
Restoration of infectivity using *in vivo* mice passages. (**A**) Scheme illustrating the sequential stages of the study. Following the collection of ‘Ld1S_luci_E2-crimson’ amastigotes from the hamster spleen, the successive *in vitro* passages of promastigotes and the infection of mice (see Fig 5B), amastigotes were recovered from the spleen of mice infected with P1 (named mP1) and from the spleen of mice infected with P21 (named mP21) at day 90 p.i., transformed in promastigotes and re-injected into naïve mice. (**B**) Parasite load kinetics in the spleen of mice infected with promastigotes P1 and P21. Lines represent the median and the colored shaded areas represent the interquartile range. The grey areas correspond to background signals (* AUCs from the infection kinetics differ significantly; P<0.05). At day 90 p.i., the mice were euthanized and the ‘persistent’ amastigotes were recovered from their respective spleens. (**CD**) Parasite load kinetics in the liver (**C**) and in the spleen (**D**) of mice infected with promastigotes mP1 and mP21, respectively. Lines represent the median and the interquartile range. The colored shaded areas represent the interquartile range of the original infection with P1 and P21 (see Fig 6B). The grey areas correspond to background signals (* AUCs from the infection kinetics differ significantly; P<0.05).

The next step was to determine whether amastigotes derived from P21-infected mice at day 90 p.i. were still able to induce an infectious process in naïve mice. To this end, we decided to collect these ‘persistent’ amastigotes of both P1 and P21 infected mice spleens and, after *in vitro* differentiation, to re-inject new naïve mice with the corresponding mP1 and mP21 parasites, and follow up the parasite load until 90 days p.i. (Fig 6A). Regardless the remarkable difference (P<0.0001) between the kinetic curves observed when mice are infected with P1 (AUC = 9.2x10^8^) and P21 (AUC = 4.9x10^7^) (Fig 6B), we observed that the subsequent infections with parasites derived from mice spleen at 90 days of infection (‘persistent’ amastigotes), either mP1 or mP21, presented similar pattern as the original infection with P1.

In the liver, the infection kinetics of mice infected with mP1 (AUC = 8.2x10^8^) was similar to the original P1 (AUC = 7.4x10^8^) infection (P = 0.5617). On the other hand, even if the parasite load of mice infected with mP21 presented lower bioluminescence values (AUC = 4.5x10^8^), it was similar to P1 (P = 0.2273) and superior than the original P21 infection (AUC = 4.0x10^7^) (Fig 6C). In the spleen, additionally, both infections with mP1 (AUC = 5.2x10^8^) and mP21 (AUC = 6.9x10^8^) originated similar infection kinetics, equivalent to P1 (P = 0.1171 and P = 0.3193, respectively) and superior than P21 (P = 0.0095 and P<0.0001, respectively) (Fig 6D).

## Discussion

New virulent *Leishmania donovani* Ld1S parasites expressing two reporter genes have been generated and validated to be used *in vitro* and *in vivo* imaging analysis. These parasites permanently express bioluminescence (firefly luciferase) and fluorescence (E2-crimson) genes, herein named Ld1S_luci_E2-crimson, and they are as virulent as wild-type parasites, as demonstrated in hamsters.

These novel parasites were key to the establishment of an innovative non-invasive experimental model of VL, which allows the real-time evaluation of the infectious process and the parasite virulence in living animals, aiming the development of longitudinal studies using the same animals. Indeed, the monitoring of the parasite load in hamsters, considered the most accepted animal model for VL [5,6], when infected with Ld1S_luci_E2-crimson, can be easily performed by bioluminescence, with the animals exhibiting a gradual but simultaneous increasing in the liver and in the spleen, accompanied by the occurrence of clinical signs such as apathy, weight loss and hepatosplenomegaly. The success of the infection can be verified in real time as soon as 15 days p.i., long before the onset of clinical signs, in a more efficient and less time-consuming manner. By contrast, when employing wild-type parasites, the effectiveness of the infection in living animals is only visible after approximately two months of infection, when the hamsters start to lose weight [5,29,30].

On the other hand, mice infected with *L*. *donovani* do not get sick, nevertheless the proposed BALB/c mouse method proved to be a reliable model to the study of VL [31–33]. The strength of this experimental model was to present an early and intense parasite implantation in the liver and in the spleen, as soon as three days post-infection[34], followed by a longstanding parasite persistence up to at least three months post-infection. All these *in vivo* bioluminescence data, validated by RT-qPCR [20,25], show that this infectious process is clearly different from other previous models using mice [8,9,35], where liver and spleen usually present distinct infection kinetics and a relative short duration of the infection, with a control of parasite load in the liver between 1 and 2 months p.i. and a delayed and low parasite burden in the spleen.

Moreover, mice actually do respond to the infection against Ld1S_luci_E2-crimson, with liver and spleen presenting a typical inflammatory response [8,33], with mixed Th1/Th2 cytokine production, as previously described in experimentally-infected hamsters [5,33], and in a similar way as the natural infection in humans and dogs [36–39]. Altogether, these features render our model exceptional, making possible global studies on parasite virulence and the quick analysis of the early infectious process.

By using Ld1S_luci_E2-crimson we are providing a new method to determine the degrees of parasite virulence. As previously shown [40], long-term *in vitro* culturing affects the virulence of *Leishmania* spp, which imposes permanent passages in animals to maintain virulent strains in the lab. Taking advantage of this characteristic, we intentionally produced attenuated *L*. *donovani* promastigotes by successive *in vitro* passages. A previous study conducted by one of us [29] ((Barja et al; in press) clearly established that virulence attenuation of parasites correlated with passage number; only promastigotes of *in vitro* passage 5 (P5) maintained virulence in hamster similar to host-derived amastigotes. Herein, we demonstrate the loss of virulence both *in vitro* and *in vivo*, by analyzing the ability of promastigotes to differentiate into amastigotes, to survive and to persist in the host’s tissues in mice determined at days 7, 30 and 90 p.i., respectively. Firstly, *in vitro* cultures clearly indicated that differences in their metabolism were observed over time, along with the increasing of the *in vitro* passage number, which included faster doubling time, in accordance with previous studies (Barja et al.; in press) and higher intensity of bioluminescence and fluorescence for the same number of parasites. Secondly, as previously shown in a hamster model [29], parasite implantation in the liver and spleen of mice was dependent on the number of *in vitro* passages. Actually, only P1 and P5 Ld1S_luci_E2-crimson promastigotes caused high implantation and long persistent infections in mice. P11 and P21 promastigotes showed a significant decrease of parasitic load in both spleen and liver at day 30 p.i., suggesting a reduction in the parasite’s capacity to persist in the organs. Interestingly, attenuated parasites (P41) were still able to implant at day 7 p.i. in host tissues, although at low intensity. Altogether, our data seem to indicate that promastigote virulence attenuation, which correlates with passage number is not due just to a deficit in implantation and differentiation into amastigotes, but also to a decrease of their capacity to persist in tissues (replication defect in amastigotes and/or control by the host immune response).

One hallmark of this study is the development of a suitable method to reinstate the virulence in a population of attenuated parasites. Despite the virulence deficit after long-term *in vitro* culturing, small numbers of parasites are still found in the organs of mice infected with these attenuated parasites. Therefore, amastigotes do not totally lose their virulence, since these parasites (herein named mP21) were still able to infect new animals and to persist in their tissues. The control of the parasite load in mice is then probably due to the progressive decrease in the number of fully virulent parasites after several passages in culture (at the promastigote level) [40,41], supporting the hypothesis that long-term *in vitro* cultures could contain a “mixture” of virulent and attenuated parasites [40], i.e., a population with an unchanged number of individuals, but with an overall reduced capacity to infect and/or persist in the tissues [42,43].

In conclusion, we described herein the generation of innovative virulent *Leishmania donovani* parasites that are concomitantly bioluminescent and fluorescent (Ld1S_luci_E2-crimson). These parasites are versatile enough to be used in a broad range of *in vitro*, *in vivo* or *ex vivo* techniques, allowing the immediate visualization of parasites or parasitized cells, such as in flow cytometry, fluorescence molecular tomography (FMT), bioluminescence assays [27,34,44]. Further, the benefits of using mice as a model of VL also include lower costs, easier handling, greater availability of reagents (such as PCR primers and antibodies). Finally, the proposed model using these novel parasites and BALB/c mice allows the non-invasive longitudinal evaluation of the infectious process in real time, as well as the parasite’s virulence and the host’s immune response. Accordingly, the results reported herein open up new perspectives on the study of new therapies and vaccines, since precise and long-lasting follow up studies are then possible, employing day-by-day verifications in a more ethical manner, without the need of frequent and numerous sample collections or euthanasia.

## Supporting information

S1 Fig *In vivo* bioluminescence evaluation in hamsters infected with *Leishmania donovani*. Hamster infected with Ld1S_luci (left, as shown in Fig 1A) and hamster infected with Ld1S_luci_E2-crimson (right) evidencing similar bioluminescent signals in both livers and spleens.
(PDF)
