# Supplementary material for: New insights into experimental visceral leishmaniasis: Real-time in vivo imaging of Leishmania donovani virulence
Source: PLoS Negl Trop Dis. 2017 Sep 25;11(9):e0005924. doi: 10.1371/journal.pntd.0005924 (PMC5629011; doi:10.1371/journal.pntd.0005924)
Supplement: S1 Fig — Hamster infected with Ld1S_luci (left, as shown in Fig 1A) and hamster infected with Ld1S_luci_E2-crimson (right) evidencing similar bioluminescent signals in both livers and spleens. (PDF) [file pntd.0005924.s001.pdf]

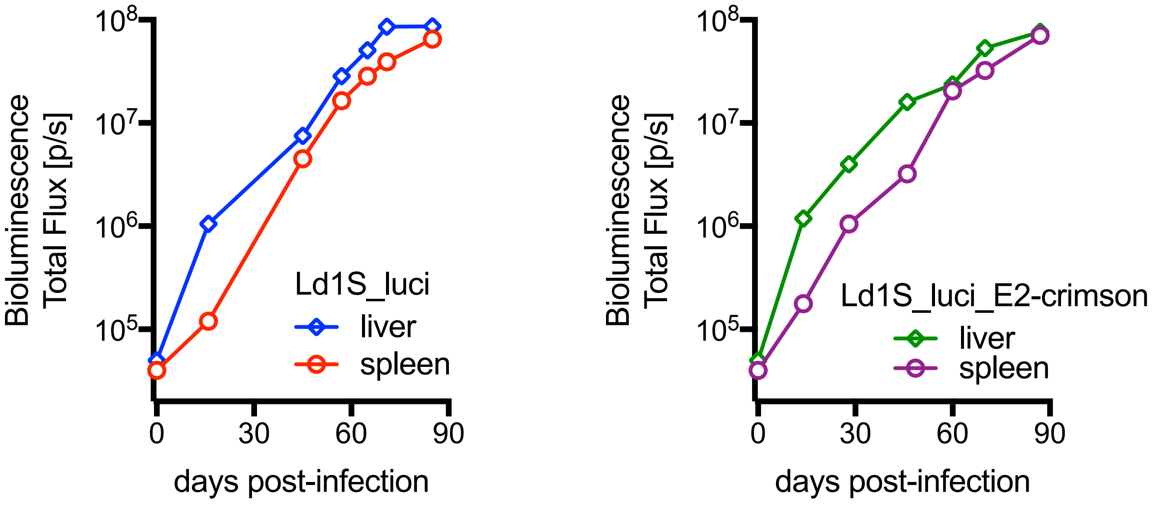

S1 FIGURE. *In vivo* bioluminescence evaluation in hamsters infected with *Leishmania donovani*. Hamster infected with Ld1S_luci (left, as shown in Figure 1A) and hamster infected with Ld1S_luci_E2-crimson (right) evidencing similar bioluminescent signals in both livers and spleens.
